# Severe CMV pneumonitis and the resulting ARDS in a 28-year-old pregnant woman: a case report

**DOI:** 10.1186/s12879-023-08091-5

**Published:** 2023-03-22

**Authors:** Sotirios Kalogeropoulos, Evgenia Zarogianni, Georgios Adonakis, Apostolos Kaponis

**Affiliations:** Department of Obstetrics and Gynecology, Patra University School of Medicine, 26504 Patra, Greece

**Keywords:** Cytomegalovirus, Pregnancy, Pneumonitis, ARDS, Severe maternal disease, Case report

## Abstract

**Background:**

Cytomegalovirus (CMV) is a common virus. In pregnant women, CMV infection is usually mildly symptomatic or asymptomatic but can lead to fetal infection. Here we present a rare case of severe CMV pneumonitis and acute respiratory distress syndrome in a healthy immunocompetent pregnant woman.

**Case presentation:**

A previous healthy 28-year-old woman with spontaneous conception, was admitted to the General University Hospital of Patras at 29 weeks of gestation with a day history of fever, fatigue, pharyngitis, and cough. She was diagnosed with acute CMV infection and CMV pneumonitis. During her hospitalization she developed acute distress syndrome (ARDS). The patient was intubated and underwent emergency caesarean delivery. She was admitted to the intensive care unit and received intravenous ganciclovir. She was discharged at 20th day postpartum in a good clinical condition.

**Conclusions:**

This case highlights the infrequent yet potential complexity of CMV infection in immunocompetent patients and in pregnancy.

## Background

Cytomegalovirus (CMV) is a very common pathogenic virus [1]. Approximately 60% of the population in developed countries and more than 90% of the population in developing countries are seropositive for CMV [2]. It is an enveloped DNA virus of the herpes viruses’ family.

The transmission of the virus occurs through body fluids and therefore with close contact, blood transfusion, organ transplantation, or vertical to the fetus. All organs can be practically infected by CMV. The majority of the immunocompetent patients with acute CMV infection will experience it asymptomatic; [3] for this reason expectant management is recommended (instead of anti-viral therapy). However, up to 7% of the cases will experience symptoms almost comparable to those of Epstein-Barr virus induced mononucleosis.

In 60–70% of pregnant women with CMV infection are symptomatic [4]. Risk of transmission for primary infection of the fetus is 30–40% in the first and second trimester, and 40–70% in the third trimester; only 10–20% of these newborns will have clinical disease at birth. Maternal infection in the first half of pregnancy is associated with an increased chance of serious manifestations in the new-born [5]. Generally, severe maternal consequences are extremely rare. Here we present a case of severe CMV pneumonitis and ARDS in a healthy pregnant woman.

## Case presentation

A 28-year-old woman with spontaneous conception, was admitted to the University Hospital of Patras at 29 weeks of gestation due to fever, fatigue, pharyngitis, and cough. Several tests were performed, including nasopharyngeal swab for SARS-CoV-2, Influenza virus antigen and RSV antigen, which were negative. The maternal serum revealed elevated CRP (C-reactive protein) (7.18 mg/dl, normal values: < 0.5 mg/dl), as well as positive both CMV IgM and IgG with low IgG avidity test (< 10%). She was seronegative for both ΗΙV I&II, HSV and EBV. The woman was also seronegative for CMV through the first and second trimester. Maternal whole blood test was also strongly positive for CMV DNA (40,917 iu/mL, positive > 178 iu/mL) by real-time polymerase chain reaction (RT-PCR). Serological immune control tests were not indicative of autoimmune disorder (ANA, p-ANCA, ASMA, RF, complement system and control for the major immunoglobulin classes). The upper abdominal ultrasound showed splenomegaly, whereas the obstetric ultrasonography did not show any fetal abnormalities.

During the first 5 days of hospitalization the patient was under observation, according to the suggestions of the infectious disease department and she was in good clinical condition. On the 6th day, the patient experienced acute chest pain and shortness of breath. She underwent a chest x-ray, which revealed a reticular pattern indicative of pneumonia. Bronco-alveolar lavage fluid was negative for bacterial superinfection. Therefore, oxygen, prophylactic anticoagulant injection, and broad spectrum antibiotics were administered (intravenous piperacillin/tazobactam) due to a further increase of inflammatory markers, CRP (15.5 mg/dl).

**Fig. 1:**
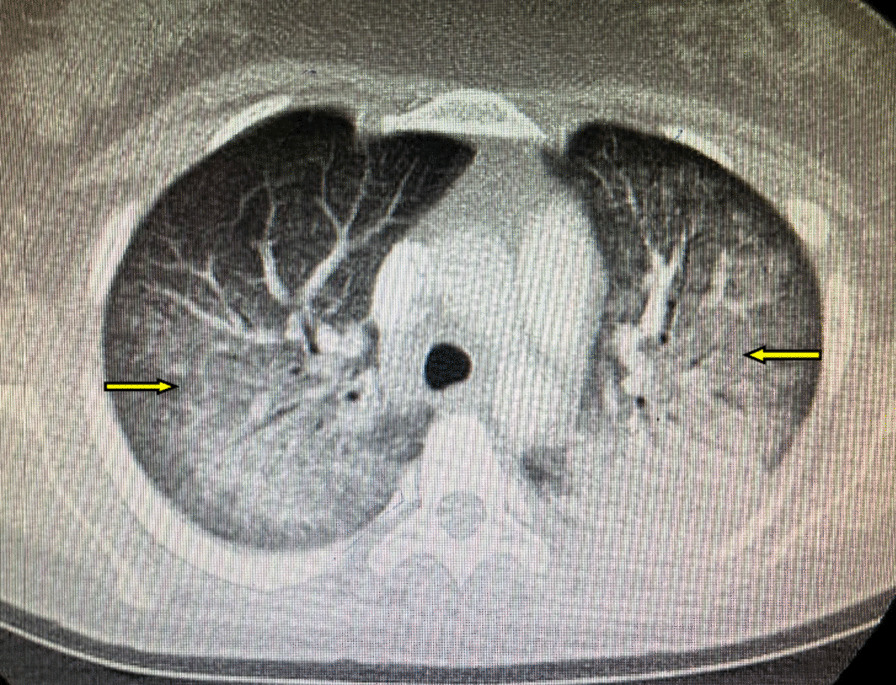
Chest CT scan demonstrating infiltrations of both lungs (arrows)

However, on the 10th day she developed progressive dyspnea, fever, and shallow breathing. She underwent a chest CT (computed tomography), which revealed infiltration of both lungs (Fig. 1). Her clinical condition deteriorated with increasing oxygen needs and respiratory failure (acute respiratory distress syndrome), so intubation was needed, and a preterm emergency cesarean section was performed at 30 weeks of gestation.

Postnatally, the patient was admitted to the intensive care unit (ICU), where she remained for 10 days and received intravenous ganciclovir (450 mg/12 h) and subsequently to the postnatal ward for another 10 days under same therapeutic scheme, after which she was discharged in a good clinical condition. She was discharged with already two negative consecutive PCR CMV DNA tests (< 178 iu/mL). Overall, ganciclovir was administered to this patient for 20 days. Meanwhile, glucocorticoids were not used during her hospitalization. The male new-born weighed 1700 gr and had an Apgar score of 6/10 at 1st min. He was intubated at 5^th^ minute, as he experienced neonatal respiratory distress syndrome and consequently was admitted to the Neonatal Intensive Care Unit. The newborn was not given antiviral agent. In admission to NICU and three times during his hospitalization CMV DNA test were sent and come back negative. (CMV DNA test through RT-PCR in both blood and urine test < 178 iu/mL). After 62 days he was discharged without any clinical signs or blood test revealing CMV infection.

## Discussion and conclusions

Neonatal and immunosuppressed patients are more likely to develop CMV complications and organ disease than immunocompetent adults. CMV organ disease in immunocompetent patients is rare but it is described and it can involve multiple organs [3, 6] (hepatitis, encephalitis, myocarditis, Guillain-Barré syndrome and pneumonitis) [7]. On the other hand, acute infection, or reactivation of CMV can be lethal in immunosuppressed patients (e.g., AIDS, congenital immunodeficiency, organ transplant recipients and other iatrogenic immunosuppression) [8].

The duration of viral DNAemia of primary CMV infection in pregnant women lasts for about 2–6 weeks [9]. In immunocompetent patients this phase resolves and leads to the latent phase of the virus infection, which lasts for life. In pregnancy, 60–70% of primary infection will result in symptoms such as fever, headache, myalgia, arthralgia, rhinitis, pharyngitis, and physical exhaustion [5].

Here we present a case of an immunocompetent pregnant woman with severe CMV pneumonitis, which led to ARDS and preterm birth. In this case no other immune dysfunction was reported. Usually, severe CMV infection appears in immunosuppressed patients. According to our knowledge, only five cases of severe CMV infection have been reported during pregnancy, [10, 11] and only one of them had severe CMV pneumonitis [12]. This is the only known case of severe CMV pneumonitis in an immunocompetent pregnant woman who survived.

The treatment of immunocompetent patients with acute CMV infection is controversial, especially during pregnancy. As in this case, symptomatic CMV infection in immunocompetent patients is considered self-limited and expectant management is recommended. On the other hand, CMV pneumonitis can be rather deadly. In the current case, the severity of the infection led to a preterm delivery via emergency Cesarian section, intubation of the patient and the use of antiviral therapy.

The complexity of viral infections during pregnancy is well known, because pregnancy is considered an immune-suppressed condition. Pregnant women are predisposed to several viral infections [13]. There are strong epidemiological evidence that pregnant women are at higher risk of severe viral infections than non-pregnant population, especially during pandemics (Influenza, Sars-cov2) [14].

There are several immunological alterations during pregnancy, which are considered physiological changes but could make pregnant women more vulnerable in infectious diseases. However, severe clinical manifestations are extremely rare, especially in CMV infection.

## Data Availability

The data, which were used in this case-report, exist in medilab-i online software lab that hosts the University General Hospital of Patras.

## Abbreviations

CMV: Cytomegalovirus
ARDS: Acute respiratory distress syndrome
CT: Computed tomography
IgM: Immunoglobulin M
IgG: Immunoglobulin G
HIV: Human immunodeficiency virus
RSV: Respiratory syncytial virus
EBV: Epstein–Barr virus
HSV: Herpes simplex virus
ANA: Antinuclear antibody
p-ANCA: Perinuclear anti-neutrophil cytoplasmic antibody
ASMA: Anti-smooth muscle antibody
RF: Rheumatoid factor
AIDS: Acquired immune deficiency syndrome
CRP: C-reactive protein
RT-PCR: Real-time polymerase chain reaction
ICU: Intensive care unit
NICU: Neonatal intensive care unit
